# Multiple choroidal osteomas in a boy – a rare presentation: a case report

**DOI:** 10.1186/s13256-019-2179-4

**Published:** 2019-08-02

**Authors:** Arup Deuri, Deepanjan Ghosh, Jayant Ekka, Vijaya Agarwalla

**Affiliations:** Department of Ophthalmology, Assam Medical College, Dibrugarh, Assam India

**Keywords:** Choroid, Osteoma, RPE, Decalcification

## Abstract

**Background:**

Choroidal osteoma is rare clinical entity of unknown etiology, characterized by formation of mature cancellous bone within the choroid. It typically affects young females, with no racial predilection. Vision loss occurs mainly due to photoreceptor degeneration secondary to decalcification and/or development of choroidal neovascularization especially if located at the subfoveal area.

**Case presentation:**

Our case is 9-year-old Indian (Indo-Aryan) boy identified incidentally with clinical features suggestive of choroidal osteoma with marked diminution of vision. Spectral domain optical coherence tomography demonstrated high reflectivity from the choroid and atrophy of the overlying retinal layers and B-scan ultrasound demonstrated multiple highly reflective calcified lesions within the choroid.

**Conclusion:**

Although available literature shows that the occurrence of this rare clinical entity is more commonly seen in young females, our case report has shown that it may be seen at a very early age. The treatment options are still not available if significant atrophy of retinal pigment epithelium has already occurred; however, vision loss due to associated choroidal neovascularization may be treated with currently available treatment options. In our case, the vision loss was due to the significant atrophy of the retinal layers. Choroidal neovascularization was not seen and our patient was advised to attend follow-up regularly.

## Background

Choroidal osteoma is a benign ossifying disorder with formation of mature cancellous bone in the choroid. The exact etiology is still unknown. The first case was presented at a meeting of the Verhoeff Society in 1975 and the case was published by Gass *et al.* in 1978 [1]. The incidence of the disease is extremely rare. No data on the prevalence are available in the literature. To date, only a few cases have been reported. The largest case series included 74 eyes of 61 patients with choroidal osteoma followed up over a period of 26 years [2]. There is no racial predilection; however, most reports were of white patients [3]. It typically affects young adults; there is a female predilection. It appears as orange-yellow to yellow-white lesions with a distinct margin with blood vessels overlying them. The lesion color depends on the level of overlying retinal pigment epithelium (RPE) depigmentation [1]. In the early stages, they tend to be orange-red in color, whereas in later stages they have a yellowish tint due to RPE depigmentation [4]. The most common causes of visual loss in these patients are due to choroidal neovascularization (CNV) and/or photoreceptor loss [5], and to choroidal and RPE atrophy associated with decalcification [2, 6, 7].

## Case presentation

**Fig. 1:**
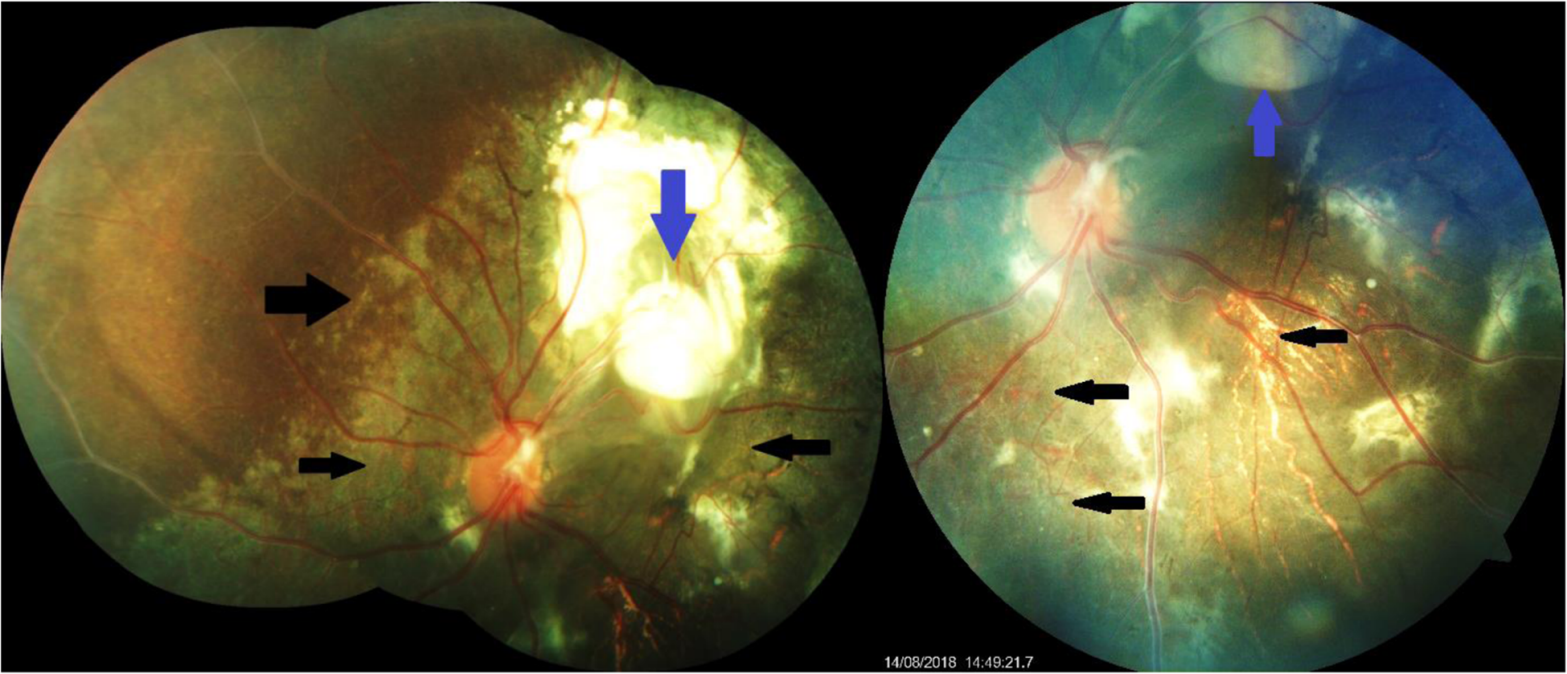
Fundus photograph of the left eye showing multiple choroidal osteomas superotemporally (*blue arrows*) and areas of retinal pigment epithelium depigmentation (*black arrows*)

A 9-year-old Indian (Indo-Aryan) boy diagnosed as having pan-sinusitis was referred to us from an Ear, Nose, and Throat (ENT) department with complaints of swelling over the left side of his face involving left lower lid to rule out any ocular manifestation. He gave a history of swelling over the left side of his face of approximately 10 days’ duration. It was insidious in onset, gradually progressive in nature, and involved the left lower lid; it was not associated with pain and fever. There was no associated systemic disease. On examination, the best corrected visual acuity (BCVA) for his left eye was hand movement and for his right eye it was 6/6. He was unaware of diminished vision in his left eye. On dilated fundus examination we observed two yellowish white lesions with well-demarcated borders located superotemporally indicative of active lesions, which were associated with nearby areas of RPE depigmentation and pigment clumps extending into the macular area, which were suggestive of degeneration of overlying retinal layers indicative of decalcified lesions in his left eye (Fig. 1). Fundus of his right eye was normal.

**Fig. 2:**
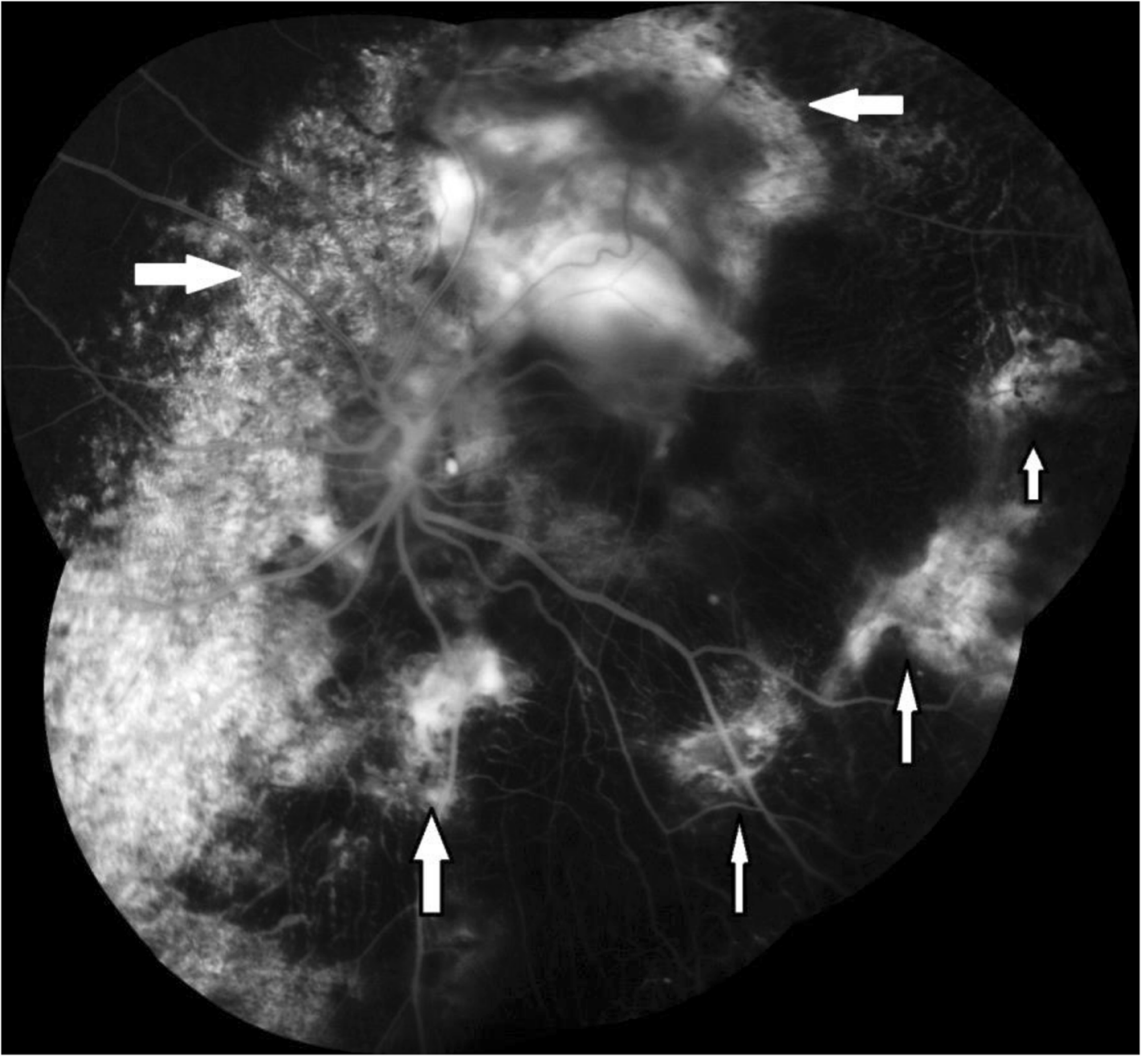
Fundus fluorescein angiography of left eye showing areas of patchy hyperfluorescence corresponding to the areas of retinal pigment epithelium depigmentation (*white arrows*)

Fundus fluorescein angiography (FFA) revealed areas of early granular hyperfluorescence corresponding to the areas of RPE depigmentation and late hyperfluorescence over the calcified lesion with some interspersed areas of hypofluorescence corresponding to the areas of pigment clumps in the left eye (Fig. 2).

**Fig. 3:**
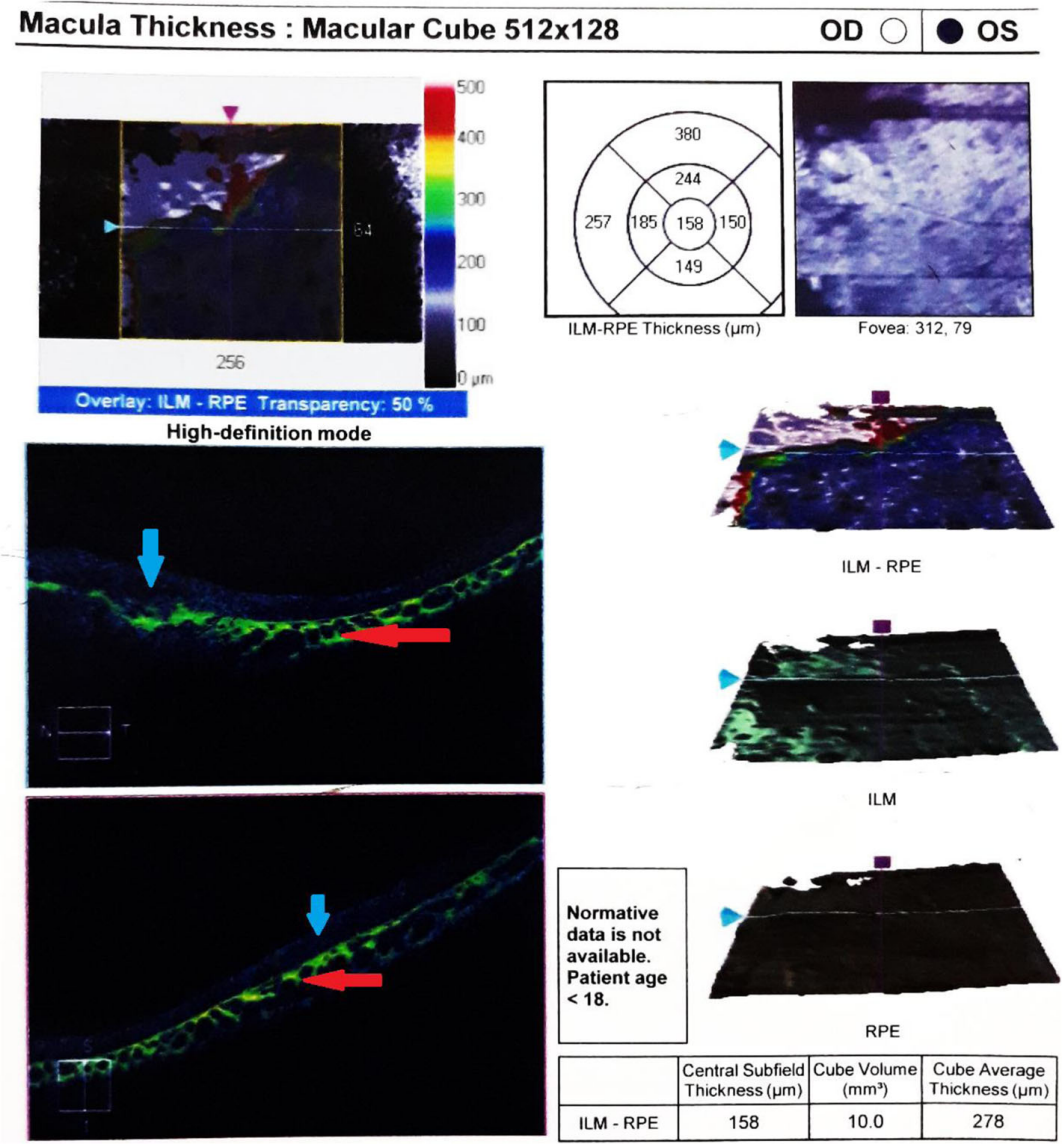
Spectral domain optical coherence tomography of macula of left eye showing high reflectivity from the choroid (*red arrows*) and thinning of the retinal layers (*blue arrows*). *ILM* inner limiting membrane, *RPE* retinal pigment epithelium

Spectral domain optical coherence tomography (SD-OCT) was performed and revealed high reflectivity from the choroid with marked thinning of overlying retinal layers including photoreceptor inner/outer segment junction (Fig. 3).

**Fig. 4:**
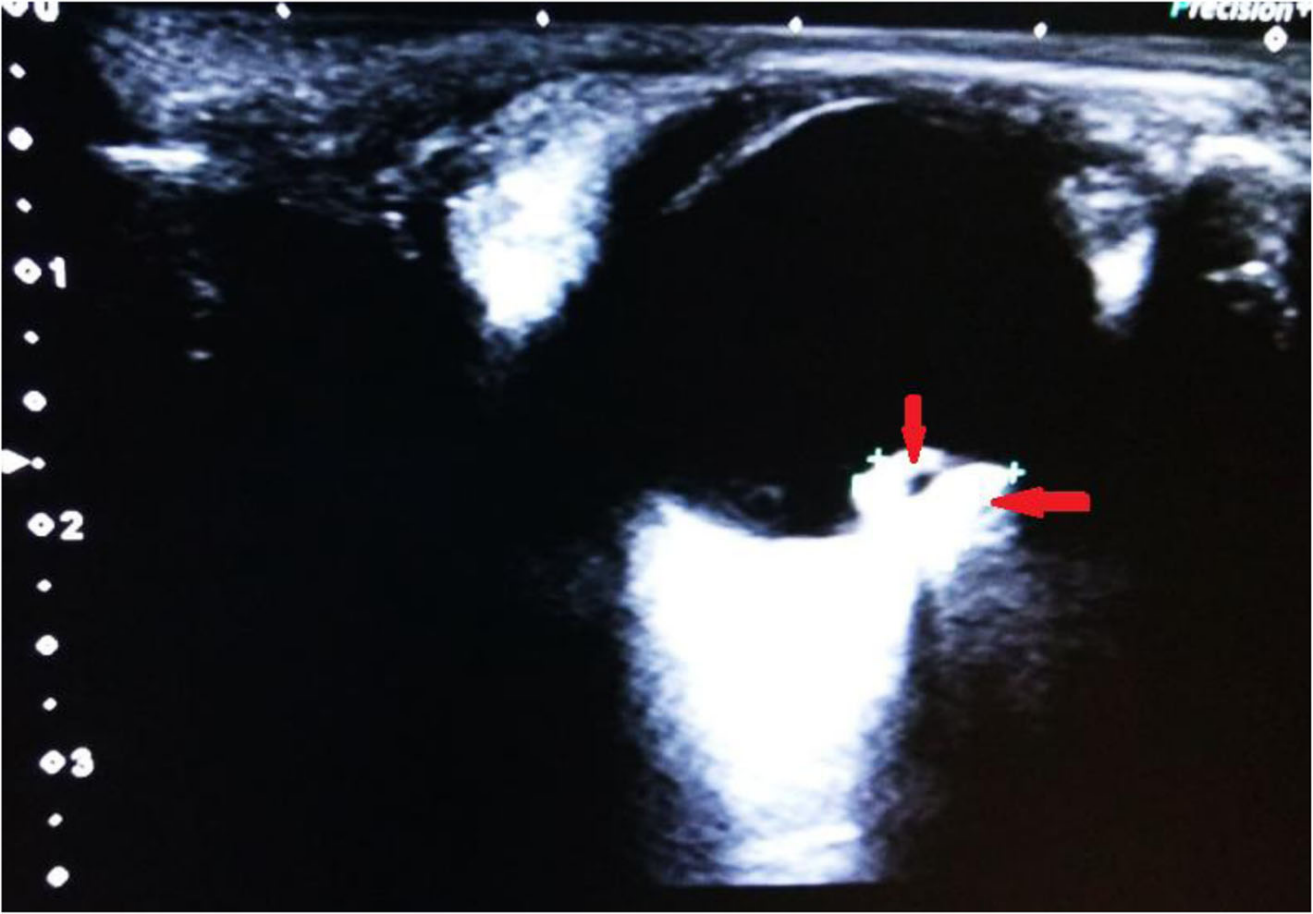
B-scan ultrasound of left eye showing echogenic calcified lesion in the choroid (*red arrows*)

B-scan ultrasound (USG) of his left eye demonstrated a large irregular echogenic calcified lesion of 7.1 × 3.9 mm in the posterior choroid near to the optic disc region and extending up to optic disc, and another smaller echogenic calcified foci in the posterolateral choroid both nasally and temporally. The nasal lesion was 1.3 mm and temporal lesion was 1.5 mm (Fig. 4).

Examination of his right eye was normal. All routine blood investigations were normal including serum calcium and parathyroid hormone level. As there was already a setup of decalcification with loss of RPE/photoreceptor and marked diminished vision, and the lesion extended into the foveal region, we asked our patient to attend follow-ups at monthly intervals to monitor the subsequent progression or regression of the tumor, because of the non-availability of a reliable treatment option.

## Discussion

Choroidal osteoma is a rare benign ossifying disorder of the choroid. In our case, our patient was a 9-year-old boy. In the largest case series on choroidal osteoma including 74 eyes of 61 patients followed up for a period of 26 years, Carol and colleagues demonstrated that choroidal osteoma is a disease of young females [2]. They found that choroidal osteoma showed evidence of growth in 51% of eyes and decalcification in nearly 50% of eyes by 10 years. In their series, decalcification of choroidal osteoma was usually associated with poor vision [2]. Decalcification was hence considered a significant risk factor for poor long-term visual acuity. Decalcification commonly occurs concurrently with overlying RPE alterations and atrophy of the choriocapillaries, both of which could lead to photoreceptor degeneration and poor visual acuity. Shields and colleagues found that the decalcified portion of osteoma displayed an overlying marked thinning to absent outer retina and photoreceptor layers (100%), compared with the calcified portion with preserved intact outer retina (95%) and intact photoreceptor layer (100%) [5]. The optical coherence tomography (OCT) findings of our case also revealed the diffuse atrophy of the outer retina on the decalcified lesion involving the macular area and explained why our patient’s vision was poor.

The etiology of choroidal osteoma is still unknown. It has been suggested that it is an osseous choristoma [8]. This suggestion is supported by peripapillary location, a site favored by other developmental tumors and by occurrence of the osteoma in the absence of any other disease process. An alternative cause is secondary ossification following inflammation or trauma to the orbit or periorbital tumor. A case of multiple osteomas developing in association with bilateral pseudotumors of the orbit raised the possibility that inflammation may have a part in the cause [9]. In our case, there was associated pan-sinusitis with multiple choroidal osteomas. Treatment options for foveal choroidal osteoma are limited. Photodynamic therapy (PDT) is a reasonable choice in the case of extrafoveal CNV lesions. Observation is the indicated management where there are no symptoms, with fundus examination at regular intervals monitoring for signs of CNV. Shields *et al.* also reported a case of extrafoveal CNV successfully treated with PDT [10]. However, the author inserted a proviso at the end of the case report that treatment of subfoveal CNV with PDT may result in worse visual acuity due to decalcification and associated RPE loss. More recently, anti-vascular endothelial growth factor (anti-VEGF) drugs have been used off license to treat CNV secondary to choroidal osteoma with good effect. In the future, more studies with long-term follow-up may help to define an appropriate time interval when intervention can be performed to prevent tumor growth or decalcification.

## Conclusion

Choroidal osteoma is a rare benign disorder of unknown etiology; the literature showed a higher incidence in young females. However, it may occur at a very early age. Treatment options are still not available; also, early detection of the condition is incidental. Most of the time, a patient presents with symptoms of diminished vision when significant atrophy of retinal layers has already occurred. If diagnosed incidentally, the patient should be followed up regularly to detect the development of CNV or atrophy of retinal layers, so that loss of vision can be minimized. The strengths of this case report include its novelty; we describe a rare disease process involving the retina, with significant reduction in vision. The workup of our patient was quite thorough, as it involved multiple hospital admissions and subspecialty evaluations. The limitations of this case report include no follow-up data, as our patient did not turn up for follow-up. Curative treatment options are not yet available for this entity.

## Data Availability

Not applicable.
